# A New Record and Description of a New Species of the Genus *Thrips*, with an Updated Key to Species from Iran

**DOI:** 10.1673/031.012.9001

**Published:** 2012-07-27

**Authors:** Majid Mirab-balou, Xiao-li Tong, Xue-xin Chen

**Affiliations:** ^1^ institute of Insect Sciences, Zhejiang University, 866 Yuhangtang Road, Hangzhou 310058, China; ^2^ Department of Entomology, College of Natural Resources & Environment, South China Agricultural University, Guangzhou 510642, China; ^3^ Present address: Postdoctoral researcher at Department of Entomology, South China Agricultural University, Guangzhou 510642, China

**Keywords:** checklist, identification

## Abstract

An illustrated key is provided to distinguish the 26 species of the genus *Thrips* L. (Thripidae: Thripinae) recorded from Iran. *Thrips alavii* Mirab-balou, Tong & Chen, **sp. n.** is described and illustrated. *Thrips alliorum* (Priesner) is newly recorded for the fauna of Iran. A checklist is provided for all recorded species in this genus from Iran, with information on the geographical distribution for each species.

## Introduction

*Thrips* L. (Thripidae: Thripinae) is the largest genus in the Thysanoptera subfamily Thripinae, with about 280 described species in the world. Most species of *Thrips* are flower-living, although a few appear to breed mainly on leaves (Mound and Ng 2009), and several species play an important role in the pollination of crops. For example, *T. hawaiiensis* (Morgan) is an effective pollinator for oil and banana palms in the pacific region, and *T. imagines* Bagnall and *T. obscuratus* (Crawford) in Australia and New Zealand (Kirk 1984). Several species of *Thrips* are considered crop pests in various parts of the world, such as *T. angusticeps* Uzel, *T. flavus* Schrank, *T. hawaiiensis* (Morgan), *T. meridionalis* Priesner, and *T. tabaci* Lindeman (Moritz et al. 2001). The latter species is well known as the most important pest of onion crops, greenhouses and ornamental plants in Iran (Khanjani & Mirab-balou 2005 a,b; Mirab-balou et al. 2008, 2009, 2012), and it is a carrier of some Tospovirus diseases on ornamental plants, especially in Tehran and Markazi provinces (Ghotbi et al. 2003). Recently, transmission of an isolate of Tomato spotted wilt virus on cineraria (*Senecio* sp.) by *T. tabaci* has been confirmed in Fars province, Iran (Rasoulpour and Izadpanah 2003).

Because species belonging to the genus *Thrips* occur worldwide, taxonomic keys for the genus have been provided for many parts of the world, i.e., California (Bailey 1957); Illinois (Stannard 1968); Korea (Woo 1974); the European part of the USSR (Dyadechko 1977); India (Bhatti 1980); Asia and Australasia (Palmer 1992); North America (Nakahara 1994; Hoddle et al. 2009); Europe and the Mediterranean (zur Strassen 2003); Pakistan (Akram et al. 2003); Australia, New Caledonia and New Zealand (Mound & Masumoto 2005); Peninsular Malaysia (Mound & Azidah 2009); Afro-tropical Region (Mound 2010); and China, including Taiwan (Han 1997; Wang 2002; Zhang et al. 2011).

Currently, 26 species of the genus *Thrips* have been recorded in Iran (Bhatti et al. 2009). A key to 72 species of *Thrips* is available in zur Strassen's book (2003), and is useful for the study of Iranian thrips. The objective of our present paper is to provide an illustrated identification key to all 26 species known from Iran, including one new species, and one new record for the fauna of this country. In addition, some important characteristics shared by Iranian species of *Thrips* are listed in Table 1; a checklist is provided for all recorded species in this genus from Iran, with information on geographical distribution for each species (Table 2). Deciding the true host plant of Thysanoptera species is difficult, because plants on which adults are found are not always the same as those on which larvae can develop. Nevertheless, thrips-associated plants in Iran are listed in Table 3.

## Materials and Methods

Thrips specimens were collected from different sites in Iran during 2007–2011. The method for preparing and mounting thrips on slides follows Mirab-balou & Chen (2010). All descriptions, measurements, and photos were made with a Leica DM IRB microscope, with a Leica Image 1000 system. All specimens were deposited in the Institute of Insect Sciences, Zhejiang University, Hangzhou, China (ZJUH). All measurements are given in micrometers, unless otherwise stated.

Specimens were also studied from the following collections: Insect Collection, Department of Entomology, South China Agricultural University, Guangzhou, Guangdong Province; and Entomological Museum, Northwest A. & F. University, Yangling, Shaanxi Province.

### Genus *Thrips* L.

All members of the genus *Thrips* lack ocellar setae pair I on the head, and they all have paired ctenidia on abdominal tergite VIII, posteromesad to the spiracles. Other characteristics, such as number of antennal segments and setae on the forewing veins, and number of discal setae on the sternites, vary between species (Palmer 1992; Nakahara 1994; Mound and Masumoto 2005; Mirabbalou and Chen 2011). See Bhatti (1980) and Mound & Masumoto (2005) for generic characteristics, and the list of its synonyms.

Among Iranian species of the genus *Thrips*, *T. tabaci* (commonly known as onion thrips or tobacco thrips) is widely distributed. This polyphagous species is particularly abundant in warm, dry sites, especially where onion, its preferred host, is grown. It is a major pest of glasshouse crops, such as cucumber, sweet pepper, chrysanthemum, and many bedding plants in Iran (Pourian et al. 2009). Taxonomically, *T. tabaci* is principally characterized by rows of ciliate microtrichia on the sides of abdominal tergites II–VII, 4–7 distal setae on the first vein of the forewing, three lateral marginal setae on abdominal tergite II, and narrow transversely elongate pore plates on sternites III–V (males only). Another species, *T. major* Uzel, is characterized by having rows of ciliate microtrichia on the sides of abdominal tergites II–VII, similar to *T. tabaci*, but it can be distinguished from the latter by the following characters: tergite VIII with comb present laterally, forewing first vein with three distal setae, and tergite IX with two pairs of campaniform sensilla. Males of this species are very rare; we found less than ten males, but more than a thousand females.

Although *T. iranicus* and *T. pistaciae* have been recorded in Iran (Bhatti et al. 2009), little information on these two species is available. Dyadechko (1977) listed several characteristics for these two species as follows: (1) antennal segment V much shorter than IV in both species; (2) forewing first vein with 6–8 distal setae in *T. pistaciae*, and 3 distal setae in *T. iranicus*; (3) antennal segments I–III brownish yellow in *T. pistaciae*, but segments I and IV dark in *T. iranicus*; (4) abdominal tergite VIII without comb on posterior margin in *T. pistaciae*.

The females of *T. trehernei* were very similar to the females of *T. physapus*, the type-species of this genus, but *T. trehernei* had abdominal tergite X more than 80 microns long (less than 80 microns long in *T. physapus*), and the major setae on the body were longer than in *T. physapus*. The two species are most readily distinguished by the males, these being brown in *T. trehernei,* and yellow in *T. physapus*.

**Key to *Thrips* species (females) in Iran**
1. Abdominal sternites with at least one pair of discal setae (Figs. 42, 43, 44) 2
— Abdominal sternites without discal setae (Fig.45) 20
2. Pleurotergites III–VII with discal setae (Fig. 11, 13, 14) 3
— Pleurorergites III–VII without discal setae (Figs. 12, 15) 12
3. Forewing first vein with five or more distal setae (Fig. 32); abdominal tergite II with 3 or 4 lateral setae 4
— Forewing first vein with three or rarely four distal setae (Figs. 7, 16, 33–35); abdominal tergite II with 3 lateral setae (Fig. 11) 7
4. Pronotum with two pairs of long setae on anterior (same as *Frankliniella*) ***T. verbasci*** (Priesner)
— Pronotum without long setae on anterior (Figs. 8– 10) 5
5. Antennae 7-segmented; abdominal tergite II with four lateral setae ***T. minutissimus*** Linnaeus
— Antennae 8-segmented (Figs. 30–31, 49–52); abdominal tergite II with 3 lateral setae (Fig. 11) 6
6. Body dark; antennal segments generally dark; abdominal sternite VII with more than 13 discal setae that arranged on two rows (Fig. 43) ***T. atratus*** Haliday
— Body blackish brown; antennal segment III pale yellow in basal third; abdominal sternite VII with about 13 discal setae that arranged in 1–2 irregular rows (Fig. 44) ***T. fraudulentus*** Priesner
7. Body yellow or light brown with a darker abdomen; antennae 7- or 8-segmented 8
— Body dark brown to black; antennae 8– segmented 9
8. Antennae 7-segmented (Fig. 39); MCS absent; median metanotal setae situated far from anterior margin ***T. pillichi* Priesner**

— Antennae 8-segmented; MCS present; median metanotal **setae** situated at **anterior** margin ***T. trybomi*** (Karny)

**9.** Metanotal campaniform sensiall (MCS) present (Fig. 18) 10
— MCS absent (Figs. 17, 19–22) 11
10. Antennal segment III light yellow, or yellow; segment VII and VIII in equal length (Fig. 50); forewing dark (Figure 16) ***T. meridionalis*** Priesner
— Antennal segment III brown to light brown (Fig. 51); segment VII about 0.6–0.7 times as length as VIII; forewing pale or shaded ***T. vulgatissimus*** Haliday
11. Postocular setae pair II small and situated well behind row; median metanotal setae situated far behind anterior margin; abdominal tergite VIII posteromarginal comb may appear, absent or represented by a few microtrichia laterally and a very short lobed flange or craspedum medially (Fig. 27) ***T. attiorum*** (Priesner)
— Postocular setae pair II in line with I & III; median metanotal setae situated near anterior margin; abdominal tergite VIII with complete comb on posterior margin (Fig. 24) ***T. alavii*** Mirab-balou, Tong & Chen, **sp. n.**

12. Abdominal sternites II–VII or III–VII with discal setae 13
— Abdominal sternites III–VI, IV–VI or V–VI with discal setae 19
13. MCS present; abdominal tergite II with four lateral setae 14
— MCS absent; abdominal tergite II with three lateral setae 15
14. Forewings with base pale; metanotum with lines of sculpture longitudinal medially, but transverse at anterior; metanotal median setae situated on anterior margin; antennae 7-or 8-segmented (Fig. 52) ***T. hawaiiensis*** (Morgan)
— Forewings pale or dark but without base distinctly paler; metanotum with sculpture broadly striate; metanotal median setae situated just behind anterior margin; antennae 7-segmented ***T. coloratus*** Schmutz
15. Antennae 8segmented ***T. simplex*** (Morison)
— Antennae 7-segmented 16
16. Forewing first vein with 5–10 (rarely with 4) distal setae (Fig. 33) ***T. angusticeps*** Uzel
— Forewing first vein with 3 distal setae 17
17. Abdominal segment X more than 80 microns long; body with long major setae ***T. trehernei*** Priesner
— Abdominal segment X less than 80 microns long; body with major setae relatively short 18
18. Antennal segment III–V and half of VI yellow; abdominal segment X usually 58–73 microns long, the sides slightly concave ***T. physapus*** Linnaeus
— Antennal segment III–V white; abdominal segment X usually 69–80 microns long, the sides straight ***T. pelikani*** Schliephake
19. Antennae 8-segmented (Fig. 49); abdominal tergite II with four lateral marginal setae ***T. vuilleti*** (Bagnall)
— Antennae 7-segmented; abdominal tergite II with three lateral marginal setae ***T. mareoticus*** (Priesner)
20. Abdominal tergite II with four lateral marginal setae (Fig. 23) 21
— Abdominal tergite II with three lateral marginal setae 22
21. Abdominal tergite VIII with complete posteromarginal comb ***T. flavus*** Schrank
— Abdominal tergite VIII with posteromarginal comb only laterally .***T. fuscipennis*** Haliday
22. Abdominal tergite VIII with posteromarginal comb laterally (Fig. 25) ***T. major*** Uzel
— Abdominal tergite VIII with complete posteromarginal comb 23
23. Abdominal tergite IX with two pair of campaniform sensilla ***T. dubius*** Priesner
— Abdominal tergite IX with one pair of campaniform sensilla (Fig. 46) 24
24. Forewings first vein with 4–7 distal setae; abdominal pleurotergites with rows of ciliate microtrichia ***T. tabaci*** Lindeman
— Forewings first vein with at most three distal setae; abdominal pleurotergal sculpture different, without closely spaced rows of microtrichia 25
25. Macropterous or micropterous (Fig. 36); body yellow ***T. nigropilosus*** Uzel
— Macropterous; body dark brown ***T. euphorbiae*** Knechtel

**Key to *Thrips* species (males) in Iran (excluding *T. alavii* and *T. fraudulentus* for which males are not known)**
1. Abdominal sternites with at least one pair of discal setae (Figs. 53–54, 56) 2
— Abdominal sternites without discal setae (Figs. 55,57–58) 17
2. Abdominal sternites III–VII with pore plate (Fig. 58) 3
— Abdominal sternites III–VI or III–V with pore plate 15
3. Pronotum with two pairs of long setae on anterior margin (same as *Frankliniella*) ***T. verbasci*** (Priesner)
— Pronotum with short setae on anterior margin 4
4. Most pleurotergites with at least one discal setae 5
— All pleurotergites without discal setae 9
5. Micropterous (Figure 37); abdominal tergite VIII with comb of a few microtrichia laterally; setae S1 on tergite IX situated anterior to S2, between campaniform sensilla, subequal in length to S2 and slightly closer together than to S2 (cf. Fig 60) ***T. attiorum*** (Priesner)
— Macropterous; other above characters variable 6
6. Antennae 7-segmented; body yellow ***T. pillichi*** Priesner
— Antennae 8-segmented; body brown to dark 7
7. Forewing first vein with 5–11 distal setae ***T. atratus*** Haliday
— Forewing first vein with 3 or rarely 4 distal setae 8
8. Body brown to dark brown ***T*.** ***vulgatissimus*** Haliday
— Body yellow ***T. trybomi*** (Karny)
9. Forewing first vein with 4 or more distal setae 10
— Forewing first vein with 3 distal setae 12
10. Antennae 8 segmented ***T. simplex*** (Morison)
— Antennae 7-segmented 11
11. Abdominal tergite IX setae S1 as length as S2; tergite VIII with incomplete posteromarginal comb ***T. angusticeps*** Uzel
— Abdominal tergite IX setae S1 slightly longer than S2; posteromarginal comb absent on tergite VIII ***T. coloratus*** Schmutz
12. Abdominal tergite II with four lateral setae; MCS present; abdominal sternites with discal setae laterally and posterior to pore plate (Fig. 56) ***T*. *hawaiiensis*** (Morgan)
— Abdominal tergite II with three lateral setae; MCS absent; abdominal sternites with discal setae laterally to pore plate 13
13. Body yellow ***T. physapus*** Linnaeus
- Body brown to dark brown 14
14. Median metanotal setae short (less than 35 microns); antennal segments IV & V pale yellow or white, VI largely yellow, only apical ⅕^th^ light brown ***T. pelikani*** Schliephake
— Median metanotal setae long (about 50 microns); antennal segments IV & V yellow basally, distally brown, segment VI brown, except basal ⅓^rd^ yellow ***T. trehernei*** Priesner
15. Abdominal sternites III–VI with pore plate; antennal segment I brown to dark brown, usually darker than II 16
— Abdominal sternites III–V (or IV) with pore plate; antennal segment I yellow or pale brown, usually as pale as II ***T. minutissimus*** Linnaeus
16. Antennae 8-segmented; MCS present ***T. meridionalis*** (Priesner)
— Antennae 7-segmented; MCS absent ***T. mareoticus*** (Priesner)
17. Abdominal sternites III–VII with pore plate (Fig. 58) 18
— Abdominal sternites III–V with pore plate (Fig. 57) ***T. tabaci*** Lindeman
18. Micropterous or brachypterous; pore plate sometimes very strongly, transversely elongated; MCS absent; abdominal tergite VIII with complete and long comb ***T. nigropilosus*** Uzel
— Macropterous; other above characters variable 19
19. MCS present ***T. fuscipennis*** Haliday
— MCS absent 20
20. Body brown; antennal segment I brown to dark brown 21
— Body yellow, or pale brown; antennal segment I white 22
21. Antennal segment V comparatively slender, 2.0–2.1 times the length as its width; pronotum and abdominal tergite IX with brown to dark brown setae (Fig. 59) ***T. dubius*** Priesner
— Antennal segment V comparatively stout, 1.5–1.7 times the length as its width; pronotum and abdominal tergite IX with pale setae ***T. euphorbiae*** Knechtel
22. Abdominal tergite II with three lateral setae *T. major* Uzel
- Abdominal tergite II with four lateral setae 23
23. Antennae 8-segmented; forewing first vein with four distal setae; body brown to dark brown *T. vuilleti* Bagnall
— Antennae 7-segmented; forewing first vein with three distal setae; body yellow *T. flavus* Schrank

**Note.** Known Iranian male *Thrips* species have pore plates on abdominal sternites as follows: on sternites III-V (or IV): *T. minutissimus*; on sternites III-V: *T. tabaci*; on sternites III–VI: *T. mareoticus* and *T. meridionalis*; and the remaining species with pore plates on sternites III–VII]

**Figures 1–7.**
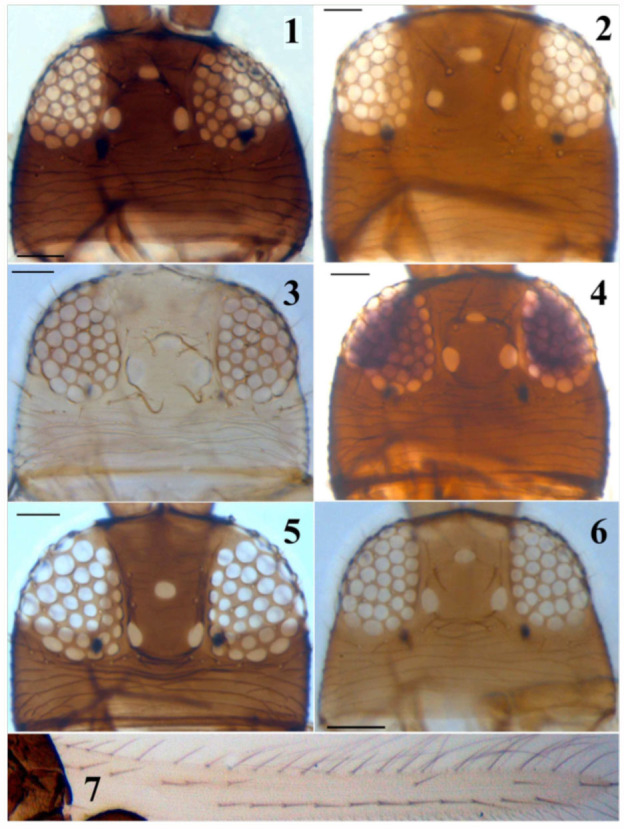
*Thrips* species. 1–6: Head: (1)*T. alavii* sp. n., (2) *T. alliorum*, (3) *T. flavus*, (4) *T. meridionalis*, (5) *T. physapus*, (6) *T. pillichi*; (7) *T. alavii* sp. n., forewing. (Scale bar = 30 microns). High quality figures are available online.

**Figures 8–16.**
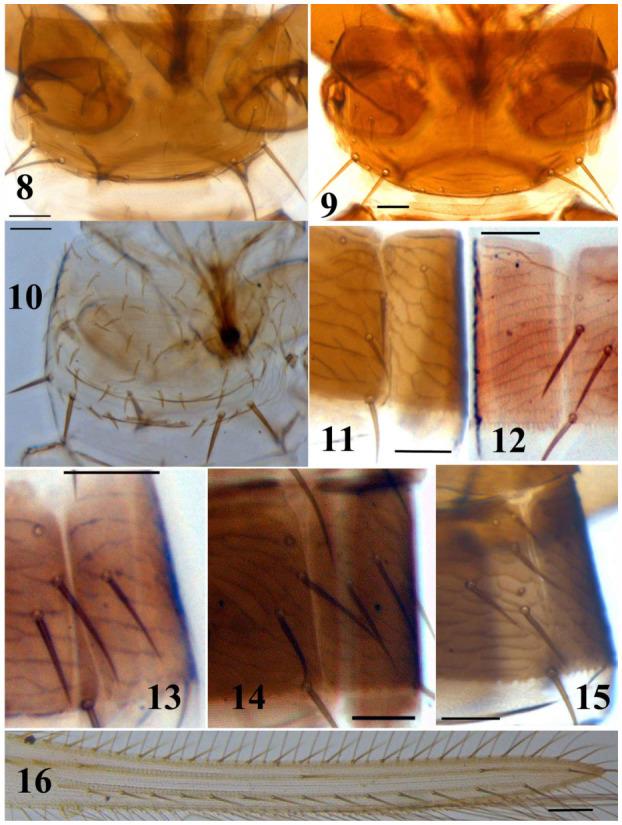
*Thrips* species. 8–9: Pronotum: (8) *T. alavii* sp. n., (9) *T. alliorum*, (10) *tabaci*; 11–15: Abdominal tergite and pleurotergite: (11) *T. alliorum*, II, (12) *T. tabaci*, V, (13) *T. alavii* sp. n., III, (14) *T. meridionalis*, V, (15) *T. hawaiiensis*, II; (16) *T. meridionalis*, forewing. (Scale bar = 30 microns). High quality figures are available online.

***Thrips alavii* Mirab-balou, Tong & Chen, sp. n. (Figures 1, 7, 8, 13, 24, 30, 42)**
**Material studied.** Holotype female (in ZJUH), Iran: Eberu (N 48° 55′, E 34° 71′, 2345 m. ASL), Hamedan Province, from *Euphorbia* sp., 8.vi.2009, Coll. M. Mirabbalou.
Description
**Female macropterous.** Body length ∼1.5 mm. Body dark brown; antennal segment III, apex of II, distal of IV and V yellowish brown, the rest uniformly brown to dark brown (Figure 30); tarsi pale brown; fore femora yellowish brown, except laterally; body setae dark brown; forewings and clavus pale (Figure 7).
**Measurements** (Slide-mounted specimens). Distended body length 1500. Head: length 170, width 120; ocellar setae III 38, II 20. Compound eyes: dorsal length 53, dorsal width 40; distance between compound eyes 53. Pronotum: median length 190, median width 125; posteroangular setae I–II 55. Forewings: length 740, hind wing 640. Abdominal tergite IX: median length 70; tergite X median length 60. Ovipositor 210. Antennal segments I to VIII had a length (width) as follows: 17 (18), 26 (16), 35 (14), 30 (11), 25 (11), 38 (11), 6 (5), and 9 (4).
**Head.** The head was 1.5 times as wide as it was long (Figure 1). The cheeks were convex, with two pairs of ocellar setae; pair III was situated outside of the ocellar triangle, and without sculpture between ocelli. Ocellar setae pair III was situated outside of ocellar triangle, behind the front ocellus. Postocular setae I & III were a little longer than others (Figure 1). The antennal was 8-segmented, with forked sense cones on antennal segments III & IV (Figure 30). Segment VI was longer than others. Antennal segments I to VIII had a length/width as follows: 0.94, 1.65, 2.66, 2.62, 2.25, 3.6, 1.5 and 2.
**Thorax.** The pronotum was 1.6 times as wide as it was long, (Figure 8); two pairs of long posteroangular setae were present; posterior margin with three pairs of setae; at least 30– 33 discal setae were present. Mesonotum with median setae far from the posterior margin; metanotum longitudinally striate (but a little more broadly striate than *T. vulgatissimus*), MCS was absent; median pair of setae were situated at the anterior margin. Mesofurca with spinula. Forewings first vein with three setae on the distal half, second vein with complete row of setae (Figure 7).
**Abdomen.** Abdominal tergites II–VIII without sculpture medially, and the median setae were small and wide apart; tergite II with 3 lateral marginal setae; tergites V–VIII with paired ctenidia laterally, on VIII posteromesad to spiracle; the comb on the posterior margin of tergite VIII was complete and long (Figure 24); pleurotergites with discal setae (Figure 13); tergite IX with two pairs of campaniform sensilla; tergite X with median slit at apex; sternites II–VII with discal setae arranged in one row (Figure 42), II with one, and III–VII with 9–11 discal setae; sternite II with two pairs of posteromarginal setae, III–VII with 3 pais; setae S1 on sternite VII arising just in front of margin. The ovipositor was well developed.
**Male.** Unknown.
**Remarks.** This new species is similar to *T. vulgatissimus*, but it is readily distinguished from the latter by the following characters: MCS absent (vs. present in *T. vulgatissimus*); metanotal median setae situated at anterior margine (vs. far behind anterior margin in *T. vulgatissimus*); abdominal sternites II–VII with discal setae that arranged in single row (vs. arranged in irregular double row in *T. vulgatissimus*). It is also distinguished from *T. alliorum* by the following character states: metanotal median setae situated near anterior margin (vs. behind anterior margin in *T. alliorum*); abdominal tergite VIII with complete comb on posterior margin (vs. may appear absent or represented by a few microtrichia laterally in *T. alliorum*); head broader than length, and postocular setae arranged in one row (vs. head elongate, and median postocular setae situated far behind rest of row in *T. alliorum*).
**Etymology.** This species is named in honor of Eng. Jalil Alavi of the Agricultural & Natural Resources Research Center of Khorasan-e-Shomali province, Bojnourd-Iran.
**Hosts.** *Euphorbia* sp. (family Euphorbiaceae).
**Distribution.** Iran: Hamedan Province.

***Thrips alliorum* (Priesner) (New record)**
*Taeniothrips alliorum* Priesner 1935: 128– 129.
*Taeniothrips carteri* Moulton 1937: 183–184.
*Thrips alliorum* (Priesner): Bhatti 1978: 195; Palmer 1992: 39–40; Han 1997: 287–289.
  This species was identified based on the descriptions by Palmer (1992), Nakahara (1994), and Han (1997), and is recorded here for the first time in Iran. This species is easily distinguished from other Iranian species by having an elongate head and median postocular setae situated far behind rest of row (Figure 2).
**Material examined.** 1,♀, Iran: Heydareh (N 48° 46′, E 34° 80′, 1968 m. ASL), Hamedan Province, from leek, 16.viii.2010, Coll. M. Mirab-balou; 1 ♀, Heydareh, Hamedan Province, from garlic, 27.vii.2010, Coll. M. Mirab-balou; deposited in the ZJUH.
**Distribution.** Iran: Hamedan Province; China, Korea, Japan, Manchuria, Hawaii (Palmer 1992; Mirab-balou et al. 2011).

**Table 1.**
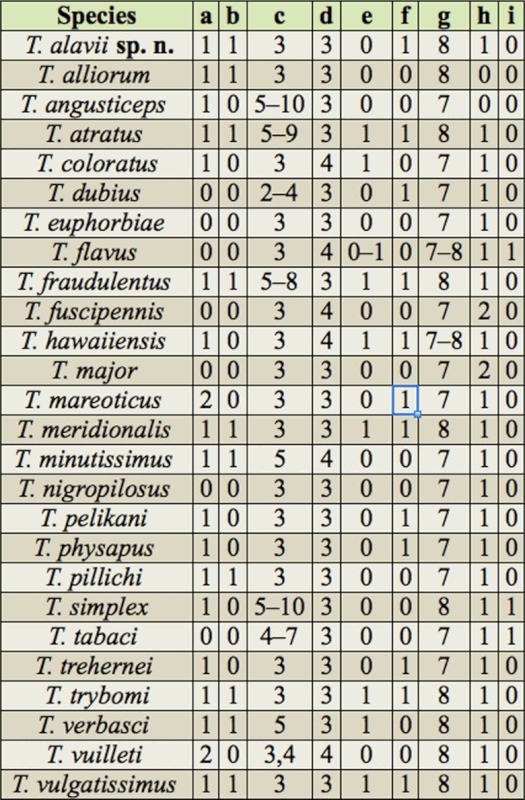
Some important characteristics of Iranian *Thrips* species. a) Discal setae on abdominal sternites: absent 0; present on sternites III–VII I ; present on sternites III–VI 2. b) Discal setae on pleurotergites: absent 0; present 1. c) Distal setae on forewing first vein, d) Number of setae on lateral tergite II. e) MCS: absent 0; present 1. f) Median metanotal setae: situated behind anterior margin 0; situated anterior at margin 1. g) Number of antennal segments, h) Abdominal tergite VIII posteromarginal comb: absent 0; present 1 ; only laterally 2. i) Position of Ocellar setae III/ocellar triangle: outside 0; inside 1.

**Table 2.**
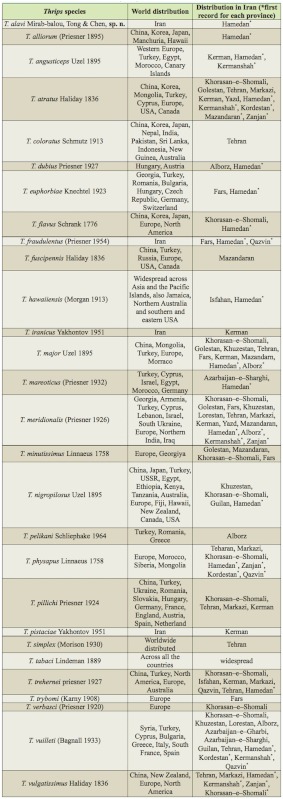
Distribution of *Thrips* species recorded from Iran.

**Table 3.**
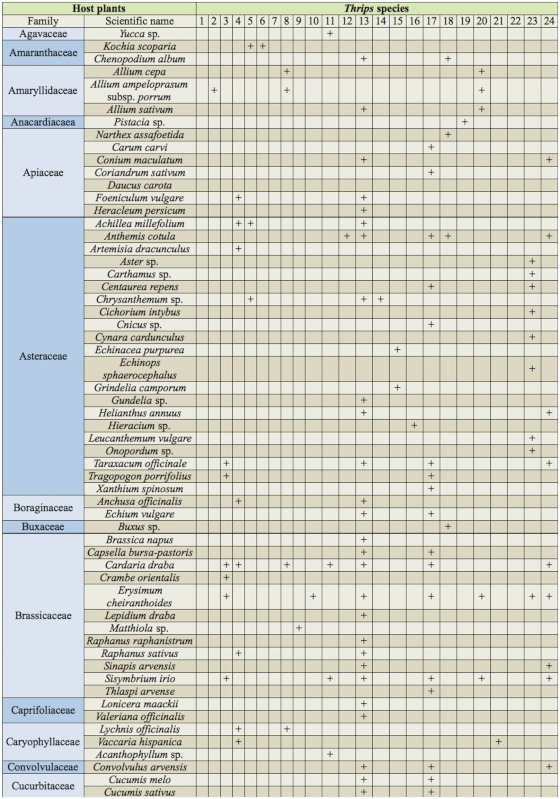
Plants in Iran associated with the genus *Thrips* (based on present study and data taken from Iranian literature).

**continued:**
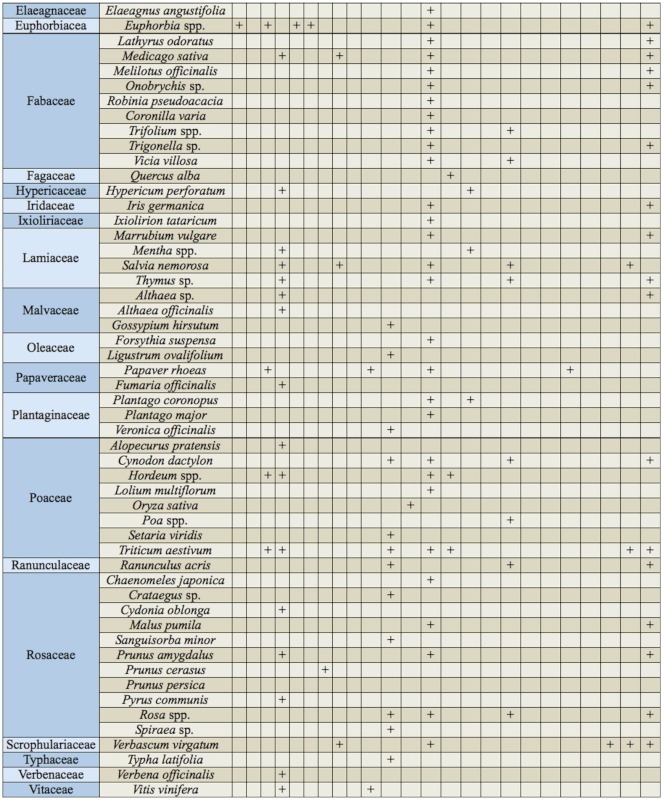

**Figures 17–23.**
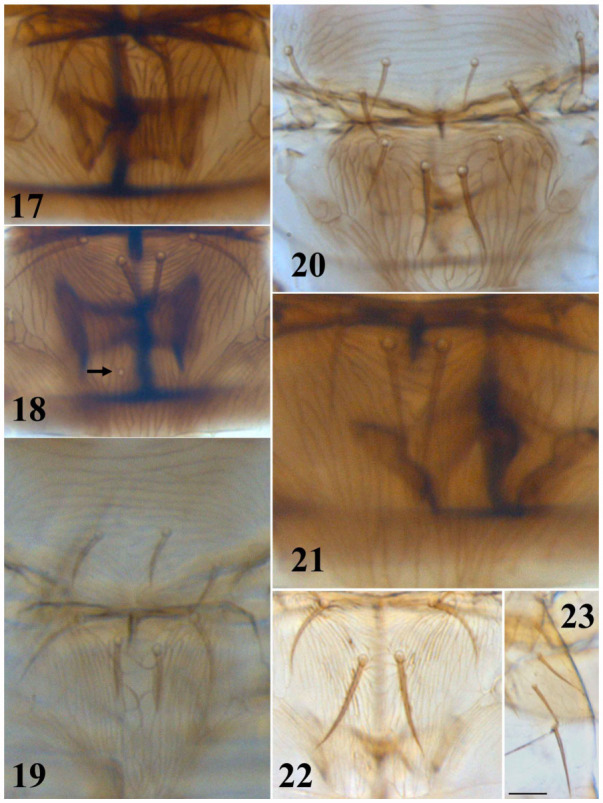
*Thrips* species. 17–22: Metanotum and meso- and metanotum: (17) *T. physapus*, (18) *T. meridionalis*, (19) *T. tabaci*, (20) *T. nigropilosus*, (21) *T. trehernei*, (22) *T. flavus*; (23) *T. flavus*, tergite II. (Scale bar = 30 microns). High quality figures are available online.

**Figures 24–31.**
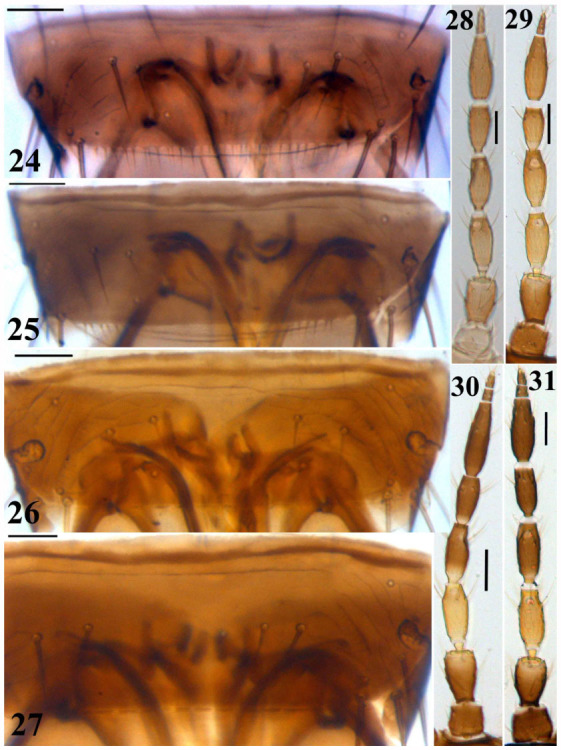
*Thrips* species. 24–27: Abdominal tergite VIII: (24) *T. alavii* sp. n., (25) *T. major,* (26) *T. angusticeps*, (27) *T. alliorum*; 28– 31 : Antennae: (28) *T. major*, (29) *T. physapus*, (30) *T. alavii* sp. n., (31) *T. alliorum*. (Scale bar = 30 microns). High quality figures are available online.

**Figures 32–41.**
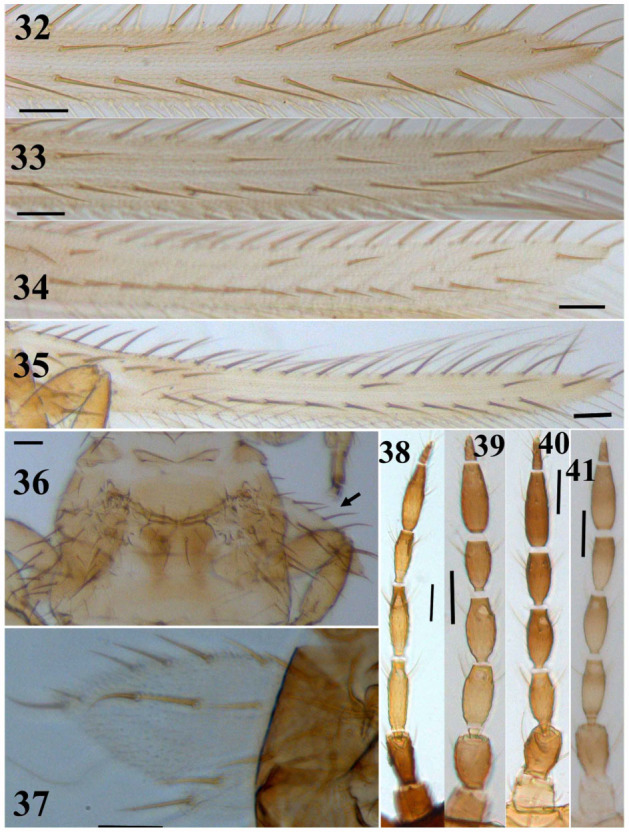
*Thrips* species. 24–27: Abdominal tergite VIII: (24) *T. alavii* sp. n., (25) *T. major*, (26) *T. angusticeps*, (27) *T. alliorum*; 28– 31 : Antennae: (28) *T. major*, (29) *T. physapus*, (30) *T. alavii* sp. n., (31) *T. alliorum*. (Scale bar = 30 microns). High quality figures are available online.

**Figures 42–52.**
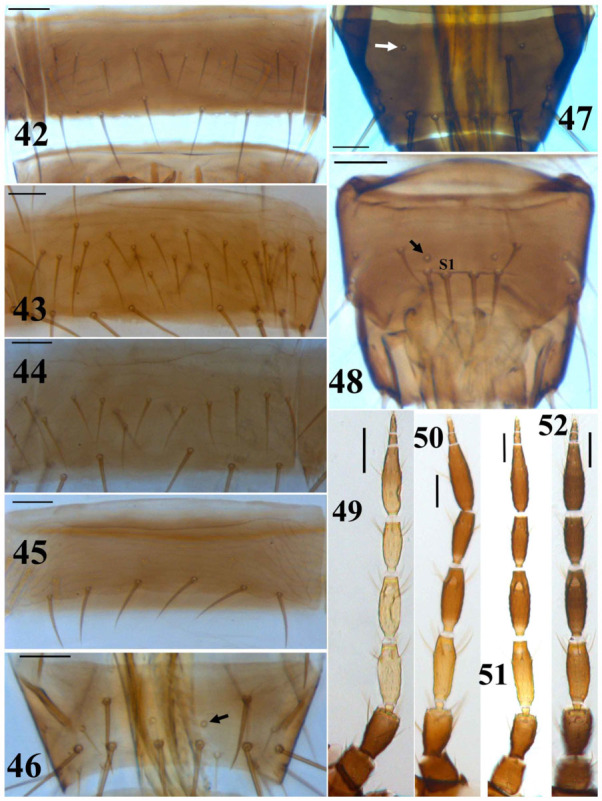
*Thrips* species. 42–45: Abdominal sternite: (42) *T. alavii* sp. n., VII, (43) *T. atratus*, VII, (44) *T. fraudulentis*, VII, (45) *T. tabaci*, Vl; 46–47: Abdominal tergite IX: (46) *T. tabaci*, (47) *T. trehernei*; (48) *T. physapus*, abdominal tergite IX, male; 49–52: Antennae: (49) *T. vuilleti*, (50) *T. meridionalis*, (51) *T. vulgatissimus*, (52) *T*. hawaiiensis. (Scale bar = 30 microns). High quality figures are available online.

**Figures 53–60.**
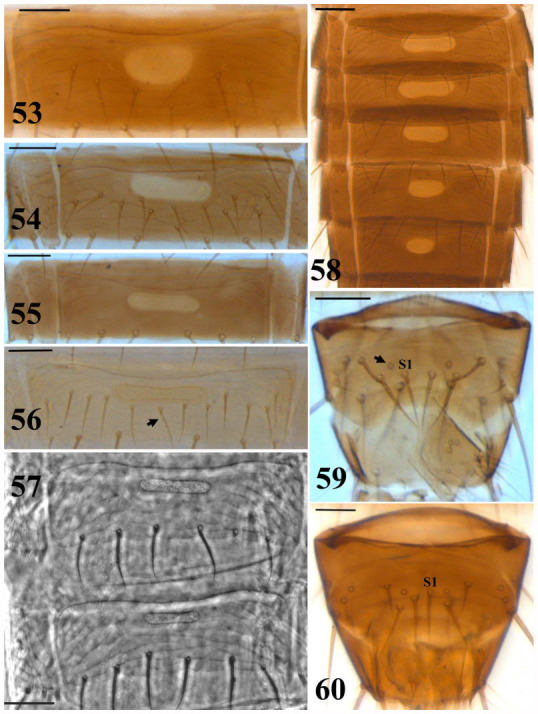
*Thrips* species. 53–58: Pore plate on abdominal sternite: (53) *T. meridionalis*, VI, (54) *T. atratus*, VII, (55) *T. dubius*, VII, (56) *T. hawaiiensis*, VI, (57) *T. tabaci*, IV–V, (58) *T*. major, III–VII; 59– 60: Abdominal tergite IX, male: (59) *T. dubius,* (60) *T. meridionalis*. (Scale bar = 30 microns). High quality figures are available online.
